# Dichlorido[2-({[3-(cyclo­hexyl­aza­nium­yl)prop­yl]imino}­meth­yl)-5-meth­oxy­phenolate]zinc

**DOI:** 10.1107/S1600536811027085

**Published:** 2011-07-13

**Authors:** Chen-Yi Wang

**Affiliations:** a Department of Chemistry, Huzhou University, Huzhou 313000, People’s Republic of China

## Abstract

The title mononuclear zinc complex, [ZnCl_2_(C_17_H_26_N_2_O_2_)], was obtained by the reaction of 2-hy­droxy-4-meth­oxy­benzaldehyde, *N*-cyclo­hexyl­propane-1,3-diamine and zinc chloride in methanol. The Zn^II^ atom is four-coordinated by the phenolate O atom and imine N atom of the bidentate zwitterionic Schiff base ligand 2-{[3-(cyclo­hexyl­amino)­prop­yl]imino­meth­yl}-5-meth­oxy­phenol, and by two chloride ions, generating a distorted ZnONCl_2_ tetra­hedral geometry. In the crystal, mol­ecules are linked by N—H⋯O hydrogen bonds, forming chains along the *c*-axis direction.

## Related literature

For the Schiff base complexes we reported previously, see: Wang (2009 ▶); Wang & Ye (2011 ▶). For similar zinc complexes, see: Zhu (2008 ▶); Wang (2007 ▶); Ikmal Hisham *et al.* (2011 ▶); Datta *et al.* (2009 ▶).
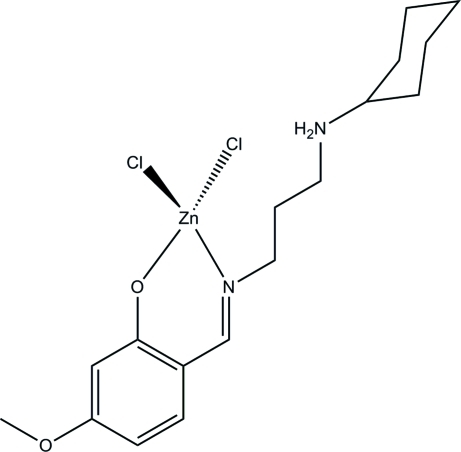


## Experimental

### 

#### Crystal data

[ZnCl_2_(C_17_H_26_N_2_O_2_)]
*M* *_r_* = 426.67
Monoclinic, 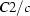

*a* = 25.118 (2) Å
*b* = 10.543 (1) Å
*c* = 14.992 (2) Å
β = 91.435 (1)°
*V* = 3968.9 (7) Å^3^
*Z* = 8
Mo *K*α radiation
μ = 1.52 mm^−1^
*T* = 298 K
0.33 × 0.30 × 0.29 mm

#### Data collection

Bruker SMART CCD diffractometer
Absorption correction: multi-scan (*SADABS*; Sheldrick, 1996 ▶) *T*_min_ = 0.634, *T*_max_ = 0.667
10824 measured reflections
4104 independent reflections
3101 reflections with *I* > 2σ(*I*)
*R*_int_ = 0.021

#### Refinement

*R*[*F* ^2^ > 2σ(*F* ^2^)] = 0.029
*wR*(*F* ^2^) = 0.075
*S* = 1.03
4104 reflections
218 parameters
H-atom parameters constrained
Δρ_max_ = 0.21 e Å^−3^
Δρ_min_ = −0.29 e Å^−3^

### 

Data collection: *SMART* (Bruker, 1998 ▶); cell refinement: *SAINT* (Bruker, 1998 ▶); data reduction: *SAINT*; program(s) used to solve structure: *SHELXS97* (Sheldrick, 2008 ▶); program(s) used to refine structure: *SHELXL97* (Sheldrick, 2008 ▶); molecular graphics: *SHELXTL* (Sheldrick, 2008 ▶); software used to prepare material for publication: *SHELXTL*.

## Supplementary Material

Crystal structure: contains datablock(s) global, I. DOI: 10.1107/S1600536811027085/hb5943sup1.cif

Structure factors: contains datablock(s) I. DOI: 10.1107/S1600536811027085/hb5943Isup2.hkl

Additional supplementary materials:  crystallographic information; 3D view; checkCIF report

## Figures and Tables

**Table 1:** Selected bond lengths (Å)

Zn1—O1	1.9554 (13)
Zn1—N1	2.0029 (17)
Zn1—Cl2	2.2129 (8)
Zn1—Cl1	2.2767 (7)

**Table 2:** Hydrogen-bond geometry (Å, °)

*D*—H⋯*A*	*D*—H	H⋯*A*	*D*⋯*A*	*D*—H⋯*A*
N2—H2*A*⋯Cl1	0.90	2.35	3.2106 (17)	160
N2—H2*B*⋯O1^i^	0.90	1.88	2.776 (2)	173

Symmetry code: (i) 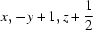.

**Fig. 1.**
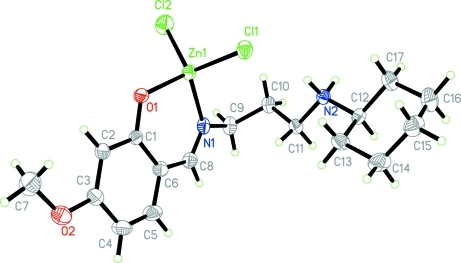
The molecular structure of the title complex, showing displacement ellipsoids drawn at the 30% probability level.

**Fig. 2.**
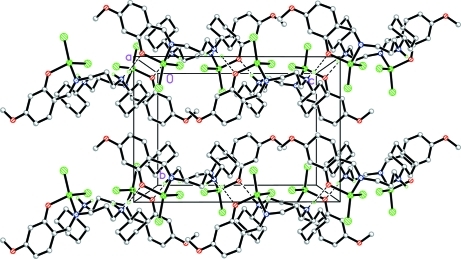
The molecular packing of the title complex, viewed along the a axis. Hydrogen bonds are drawn as dashed lines. H atoms not related to the hydrogen bonding are omitted.

## supplementary crystallographic information

### Comment

As part of our investigations into Schiff base complexes (Wang & Ye, 2011; Wang, 2009), we have synthesized the title compound, a new mononuclear zinc(II) complex, Fig. 1. The Zn atom in the complex is four-coordinated by one phenolate O and one imine N atoms of the Schiff base ligand 2-[(3-cyclohexylaminopropylimino)methyl]-5-methoxyphenol, and by two Cl atoms, generating a distorted tetrahedral geometry. The Zn—O, Zn—N, and Zn—Cl bond lengths (Table 1) are comparable with those observed in other similar zinc(II) complexes (Zhu, 2008; Wang, 2007; Ikmal Hisham *et al.*, 2011; Datta *et al.*, 2009). In the crystal, molecules are linked *via* intermolecular N—H···O hydrogen bonds (Table 2), forming chains along the *c* direction (Fig. 2)

### Experimental

2-Hydroxy-4-methoxybenzaldehyde (1.0 mmol, 0.152 g), *N*-cyclohexylpropane-1,3-diamine (1.0 mmol, 0.156 g), and zinc chloride (1.0 mmol, 0.137 g) were dissolved in MeOH (30 ml). The mixture was stirred at room temperature for 10 min to give a clear colorless solution. After keeping the solution in air for several days, colorless block-shaped crystals were formed at the bottom of the vessel.

### Refinement

All H atoms were placed in geometrically idealized positions and constrained to ride on their parent atoms, with C—H distances in the range 0.93–0.97 Å, N—H distances of 0.90 Å, and with *U*_iso_(H) set at 1.2*U*_eq_(C,*N*) and 1.5*U*_eq_(methyl C).

### Crystal data

**Table:**

[ZnCl_2_(C_17_H_26_N_2_O_2_)]	*F*(000) = 1776
*M**_r_* = 426.67	*D*_x_ = 1.428 Mg m^−^^3^
Monoclinic, *C*2/*c*	Mo *K*α radiation, λ = 0.71073 Å
*a* = 25.118 (2) Å	Cell parameters from 3616 reflections
*b* = 10.543 (1) Å	θ = 2.5–26.9°
*c* = 14.992 (2) Å	µ = 1.52 mm^−^^1^
β = 91.435 (1)°	*T* = 298 K
*V* = 3968.9 (7) Å^3^	Block, colorless
*Z* = 8	0.33 × 0.30 × 0.29 mm

### Data collection

**Table:**

Bruker SMART CCD diffractometer	4104 independent reflections
Radiation source: fine-focus sealed tube	3101 reflections with *I* > 2σ(*I*)
graphite	*R*_int_ = 0.021
ω scans	θ_max_ = 26.5°, θ_min_ = 2.1°
Absorption correction: multi-scan (*SADABS*; Sheldrick, 1996)	*h* = −31→30
*T*_min_ = 0.634, *T*_max_ = 0.667	*k* = −8→13
10824 measured reflections	*l* = −18→18

### Refinement

**Table:**

Refinement on *F*^2^	Primary atom site location: structure-invariant direct methods
Least-squares matrix: full	Secondary atom site location: difference Fourier map
*R*[*F*^2^ > 2σ(*F*^2^)] = 0.029	Hydrogen site location: inferred from neighbouring sites
*wR*(*F*^2^) = 0.075	H-atom parameters constrained
*S* = 1.03	*w* = 1/[σ^2^(*F*_o_^2^) + (0.0333*P*)^2^ + 1.6888*P*] where *P* = (*F*_o_^2^ + 2*F*_c_^2^)/3
4104 reflections	(Δ/σ)_max_ < 0.001
218 parameters	Δρ_max_ = 0.21 e Å^−^^3^
0 restraints	Δρ_min_ = −0.29 e Å^−^^3^

### Special details

**Table:**

Geometry. All esds (except the esd in the dihedral angle between two l.s. planes) are estimated using the full covariance matrix. The cell esds are taken into account individually in the estimation of esds in distances, angles and torsion angles; correlations between esds in cell parameters are only used when they are defined by crystal symmetry. An approximate (isotropic) treatment of cell esds is used for estimating esds involving l.s. planes.
Refinement. Refinement of F^2^ against ALL reflections. The weighted R-factor wR and goodness of fit S are based on F^2^, conventional R-factors R are based on F, with F set to zero for negative F^2^. The threshold expression of F^2^ > 2sigma(F^2^) is used only for calculating R-factors(gt) etc. and is not relevant to the choice of reflections for refinement. R-factors based on F^2^ are statistically about twice as large as those based on F, and R- factors based on ALL data will be even larger.

### Fractional atomic coordinates and isotropic or equivalent isotropic displacement parameters (Å^2^)

**Table:**

	*x*	*y*	*z*	*U*_iso_*/*U*_eq_	
Zn1	0.159066 (10)	0.50173 (2)	0.107303 (15)	0.04105 (9)	
Cl1	0.08297 (2)	0.50677 (5)	0.18660 (4)	0.04897 (14)	
Cl2	0.19579 (3)	0.68984 (6)	0.08640 (4)	0.06432 (18)	
N1	0.20745 (6)	0.36504 (17)	0.15451 (10)	0.0402 (4)	
N2	0.11696 (6)	0.38198 (15)	0.37490 (10)	0.0352 (4)	
H2A	0.0999	0.4227	0.3298	0.042*	
H2B	0.1262	0.4405	0.4162	0.042*	
O1	0.14586 (6)	0.42308 (13)	−0.00928 (8)	0.0435 (3)	
O2	0.12094 (6)	0.07054 (15)	−0.20497 (11)	0.0587 (4)	
C1	0.15368 (7)	0.30174 (18)	−0.02645 (12)	0.0344 (4)	
C2	0.13357 (7)	0.25212 (19)	−0.10742 (13)	0.0381 (5)	
H2	0.1160	0.3056	−0.1477	0.046*	
C3	0.13941 (8)	0.1257 (2)	−0.12835 (14)	0.0429 (5)	
C4	0.16610 (10)	0.0448 (2)	−0.06927 (17)	0.0562 (6)	
H4	0.1695	−0.0409	−0.0826	0.067*	
C5	0.18707 (9)	0.0919 (2)	0.00792 (16)	0.0553 (6)	
H5	0.2055	0.0371	0.0462	0.066*	
C6	0.18229 (8)	0.2201 (2)	0.03293 (13)	0.0399 (5)	
C7	0.09238 (12)	0.1469 (3)	−0.26836 (17)	0.0747 (8)	
H7A	0.0606	0.1786	−0.2421	0.112*	
H7B	0.0831	0.0968	−0.3199	0.112*	
H7C	0.1143	0.2169	−0.2858	0.112*	
C8	0.20912 (8)	0.2578 (2)	0.11472 (14)	0.0443 (5)	
H8	0.2304	0.1963	0.1422	0.053*	
C9	0.23994 (8)	0.3838 (2)	0.23653 (13)	0.0476 (5)	
H9A	0.2670	0.4472	0.2260	0.057*	
H9B	0.2577	0.3050	0.2525	0.057*	
C10	0.20563 (8)	0.4261 (2)	0.31269 (13)	0.0431 (5)	
H10A	0.1861	0.5017	0.2948	0.052*	
H10B	0.2284	0.4479	0.3636	0.052*	
C11	0.16647 (8)	0.3247 (2)	0.34013 (14)	0.0416 (5)	
H11A	0.1576	0.2716	0.2891	0.050*	
H11B	0.1829	0.2715	0.3858	0.050*	
C12	0.07854 (7)	0.29055 (19)	0.41592 (13)	0.0377 (4)	
H12	0.0977	0.2403	0.4615	0.045*	
C13	0.05542 (8)	0.2016 (2)	0.34577 (15)	0.0494 (5)	
H13A	0.0838	0.1523	0.3201	0.059*	
H13B	0.0383	0.2506	0.2983	0.059*	
C14	0.01487 (10)	0.1127 (2)	0.38684 (19)	0.0663 (7)	
H14A	−0.0010	0.0598	0.3404	0.080*	
H14B	0.0329	0.0575	0.4297	0.080*	
C15	−0.02850 (9)	0.1850 (2)	0.43294 (18)	0.0616 (7)	
H15A	−0.0520	0.1258	0.4619	0.074*	
H15B	−0.0494	0.2324	0.3890	0.074*	
C16	−0.00489 (9)	0.2753 (3)	0.50150 (17)	0.0676 (8)	
H16A	0.0127	0.2271	0.5488	0.081*	
H16B	−0.0332	0.3243	0.5276	0.081*	
C17	0.03513 (8)	0.3651 (2)	0.46006 (15)	0.0511 (6)	
H17A	0.0171	0.4186	0.4162	0.061*	
H17B	0.0507	0.4194	0.5060	0.061*	

### Atomic displacement parameters (Å^2^)

**Table:**

	*U*^11^	*U*^22^	*U*^33^	*U*^12^	*U*^13^	*U*^23^
Zn1	0.04827 (15)	0.04339 (15)	0.03132 (13)	0.00342 (11)	−0.00238 (10)	−0.00043 (10)
Cl1	0.0447 (3)	0.0621 (4)	0.0400 (3)	0.0052 (3)	−0.0005 (2)	0.0051 (3)
Cl2	0.0750 (4)	0.0583 (4)	0.0596 (4)	−0.0202 (3)	0.0004 (3)	−0.0009 (3)
N1	0.0339 (9)	0.0555 (11)	0.0311 (9)	0.0034 (8)	0.0014 (7)	0.0037 (8)
N2	0.0375 (9)	0.0386 (9)	0.0292 (8)	−0.0036 (7)	−0.0023 (7)	−0.0006 (7)
O1	0.0626 (9)	0.0365 (8)	0.0311 (7)	0.0094 (7)	−0.0061 (6)	−0.0007 (6)
O2	0.0749 (11)	0.0453 (9)	0.0555 (10)	−0.0138 (8)	−0.0046 (8)	−0.0102 (8)
C1	0.0317 (10)	0.0370 (11)	0.0348 (10)	0.0020 (8)	0.0068 (8)	0.0028 (8)
C2	0.0362 (10)	0.0412 (11)	0.0371 (11)	−0.0016 (9)	0.0029 (8)	0.0010 (9)
C3	0.0406 (11)	0.0427 (12)	0.0456 (12)	−0.0061 (10)	0.0064 (9)	−0.0047 (10)
C4	0.0659 (15)	0.0373 (12)	0.0655 (16)	0.0064 (11)	−0.0001 (13)	−0.0046 (12)
C5	0.0576 (14)	0.0468 (14)	0.0611 (15)	0.0142 (11)	−0.0058 (12)	0.0048 (12)
C6	0.0345 (10)	0.0442 (12)	0.0411 (11)	0.0065 (9)	0.0006 (9)	0.0017 (9)
C7	0.098 (2)	0.0668 (17)	0.0577 (16)	−0.0215 (16)	−0.0208 (15)	−0.0052 (14)
C8	0.0368 (11)	0.0531 (14)	0.0432 (12)	0.0113 (10)	0.0016 (9)	0.0088 (11)
C9	0.0319 (11)	0.0718 (16)	0.0389 (11)	−0.0018 (10)	−0.0018 (9)	0.0049 (11)
C10	0.0385 (11)	0.0552 (13)	0.0353 (11)	−0.0071 (10)	−0.0018 (9)	−0.0021 (10)
C11	0.0406 (11)	0.0445 (12)	0.0401 (11)	0.0029 (9)	0.0071 (9)	−0.0012 (9)
C12	0.0353 (10)	0.0423 (11)	0.0354 (10)	−0.0025 (9)	−0.0010 (8)	0.0064 (9)
C13	0.0473 (12)	0.0404 (12)	0.0607 (14)	−0.0069 (10)	0.0071 (11)	−0.0095 (11)
C14	0.0581 (15)	0.0444 (14)	0.097 (2)	−0.0137 (12)	0.0108 (14)	0.0005 (14)
C15	0.0444 (13)	0.0591 (15)	0.0817 (18)	−0.0114 (12)	0.0087 (12)	0.0103 (14)
C16	0.0505 (14)	0.089 (2)	0.0642 (16)	−0.0095 (13)	0.0186 (12)	−0.0027 (14)
C17	0.0425 (12)	0.0611 (15)	0.0501 (13)	−0.0067 (11)	0.0076 (10)	−0.0128 (11)

### Geometric parameters (Å, °)

**Table:**

Zn1—O1	1.9554 (13)	C8—H8	0.9300
Zn1—N1	2.0029 (17)	C9—C10	1.515 (3)
Zn1—Cl2	2.2129 (8)	C9—H9A	0.9700
Zn1—Cl1	2.2767 (7)	C9—H9B	0.9700
N1—C8	1.279 (3)	C10—C11	1.516 (3)
N1—C9	1.472 (2)	C10—H10A	0.9700
N2—C11	1.488 (2)	C10—H10B	0.9700
N2—C12	1.506 (2)	C11—H11A	0.9700
N2—H2A	0.9000	C11—H11B	0.9700
N2—H2B	0.9000	C12—C17	1.510 (3)
O1—C1	1.320 (2)	C12—C13	1.514 (3)
O2—C3	1.359 (2)	C12—H12	0.9800
O2—C7	1.426 (3)	C13—C14	1.526 (3)
C1—C2	1.404 (3)	C13—H13A	0.9700
C1—C6	1.421 (3)	C13—H13B	0.9700
C2—C3	1.378 (3)	C14—C15	1.511 (3)
C2—H2	0.9300	C14—H14A	0.9700
C3—C4	1.390 (3)	C14—H14B	0.9700
C4—C5	1.354 (3)	C15—C16	1.511 (3)
C4—H4	0.9300	C15—H15A	0.9700
C5—C6	1.408 (3)	C15—H15B	0.9700
C5—H5	0.9300	C16—C17	1.525 (3)
C6—C8	1.440 (3)	C16—H16A	0.9700
C7—H7A	0.9600	C16—H16B	0.9700
C7—H7B	0.9600	C17—H17A	0.9700
C7—H7C	0.9600	C17—H17B	0.9700
			
O1—Zn1—N1	95.66 (6)	H9A—C9—H9B	108.1
O1—Zn1—Cl2	108.39 (5)	C9—C10—C11	112.44 (18)
N1—Zn1—Cl2	116.33 (5)	C9—C10—H10A	109.1
O1—Zn1—Cl1	110.54 (5)	C11—C10—H10A	109.1
N1—Zn1—Cl1	110.01 (5)	C9—C10—H10B	109.1
Cl2—Zn1—Cl1	114.29 (3)	C11—C10—H10B	109.1
C8—N1—C9	118.95 (18)	H10A—C10—H10B	107.8
C8—N1—Zn1	119.85 (14)	N2—C11—C10	111.25 (17)
C9—N1—Zn1	121.17 (14)	N2—C11—H11A	109.4
C11—N2—C12	115.66 (16)	C10—C11—H11A	109.4
C11—N2—H2A	108.4	N2—C11—H11B	109.4
C12—N2—H2A	108.4	C10—C11—H11B	109.4
C11—N2—H2B	108.4	H11A—C11—H11B	108.0
C12—N2—H2B	108.4	N2—C12—C17	108.84 (17)
H2A—N2—H2B	107.4	N2—C12—C13	110.56 (16)
C1—O1—Zn1	124.22 (12)	C17—C12—C13	110.99 (16)
C3—O2—C7	118.51 (18)	N2—C12—H12	108.8
O1—C1—C2	118.52 (17)	C17—C12—H12	108.8
O1—C1—C6	122.71 (17)	C13—C12—H12	108.8
C2—C1—C6	118.77 (18)	C12—C13—C14	110.40 (19)
C3—C2—C1	121.28 (19)	C12—C13—H13A	109.6
C3—C2—H2	119.4	C14—C13—H13A	109.6
C1—C2—H2	119.4	C12—C13—H13B	109.6
O2—C3—C2	124.8 (2)	C14—C13—H13B	109.6
O2—C3—C4	115.2 (2)	H13A—C13—H13B	108.1
C2—C3—C4	120.1 (2)	C15—C14—C13	111.7 (2)
C5—C4—C3	119.4 (2)	C15—C14—H14A	109.3
C5—C4—H4	120.3	C13—C14—H14A	109.3
C3—C4—H4	120.3	C15—C14—H14B	109.3
C4—C5—C6	123.1 (2)	C13—C14—H14B	109.3
C4—C5—H5	118.5	H14A—C14—H14B	107.9
C6—C5—H5	118.5	C14—C15—C16	110.7 (2)
C5—C6—C1	117.37 (19)	C14—C15—H15A	109.5
C5—C6—C8	116.79 (19)	C16—C15—H15A	109.5
C1—C6—C8	125.77 (19)	C14—C15—H15B	109.5
O2—C7—H7A	109.5	C16—C15—H15B	109.5
O2—C7—H7B	109.5	H15A—C15—H15B	108.1
H7A—C7—H7B	109.5	C15—C16—C17	111.4 (2)
O2—C7—H7C	109.5	C15—C16—H16A	109.3
H7A—C7—H7C	109.5	C17—C16—H16A	109.3
H7B—C7—H7C	109.5	C15—C16—H16B	109.3
N1—C8—C6	128.39 (19)	C17—C16—H16B	109.3
N1—C8—H8	115.8	H16A—C16—H16B	108.0
C6—C8—H8	115.8	C12—C17—C16	110.3 (2)
N1—C9—C10	110.81 (16)	C12—C17—H17A	109.6
N1—C9—H9A	109.5	C16—C17—H17A	109.6
C10—C9—H9A	109.5	C12—C17—H17B	109.6
N1—C9—H9B	109.5	C16—C17—H17B	109.6
C10—C9—H9B	109.5	H17A—C17—H17B	108.1

### Hydrogen-bond geometry (Å, °)

**Table:**

*D*—H···*A*	*D*—H	H···*A*	*D*···*A*	*D*—H···*A*
N2—H2A···Cl1	0.90	2.35	3.2106 (17)	160
N2—H2B···O1^i^	0.90	1.88	2.776 (2)	173

Symmetry codes: (i) *x*, −*y*+1, *z*+1/2.
